# A Rare Case of Spontaneous Empyema by *Clostridium perfringens*

**DOI:** 10.1155/2018/2791349

**Published:** 2018-01-08

**Authors:** Sijan Basnet, Izza Mir, Elan Mohanty, Rashmi Dhital, Biswaraj Tharu, Dilli Ram Poudel

**Affiliations:** ^1^Department of Medicine, Reading Health System, West Reading, PA 19611, USA; ^2^Maharajgunj Medical Campus, Tribhuvan University, Kathmandu, Nepal

## Abstract

Empyema caused by clostridial infections is rare especially in the absence of invasive thoracic procedures. We present the case of an 81-year-old man without a history of preceding trauma who presented with shortness of breath and nonproductive cough and grew *Clostridium perfringens* only in the pleural cavity. He was predisposed to the infection due to his swallowing dysfunction. He was treated with penicillin and chest tube placement for drainage and subsequently improved.

## 1. Introduction

Clostridial infections of the lung and pleura are uncommon [1, 2]. These infections are usually reported in the event of trauma, invasive percutaneous procedures of the pleural cavity, or chronic disease or aspiration [1, 3, 4]. Spontaneous cases of *Clostridium perfringens* in the absence of abovementioned risk factors are rare [1]. We report the case of an 81-year-old man with *C. perfringens* empyema.

## 2. Case Description

An 81-year-old man presented to the emergency department (ED) with shortness of breath and nonproductive cough. He denied fever, chills, or chest pain. He was afebrile with blood pressure 127/68 mmHg, pulse rate 63/min, and respiratory rate 22/min. The patient's saturation was 89% in room air which improved to 97% with 2L of oxygen. He had decreased breath sounds with crackles over the right lung field. The remainder of the physical exam was unremarkable. The patient had been admitted 2 weeks before with lower extremity weakness which was thought to be secondary to deconditioning from a recent upper respiratory tract infection for which he was treated with amoxicillin by his primary care physician. At that time, he was discharged with provisions for physical therapy. The patient had a past medical history of paroxysmal atrial fibrillation on amiodarone but not on anticoagulation, hypertension, and chronic kidney disease stage III. He had a 20 pack-year smoking history and had quit 57 years ago.

**Figure 1:**
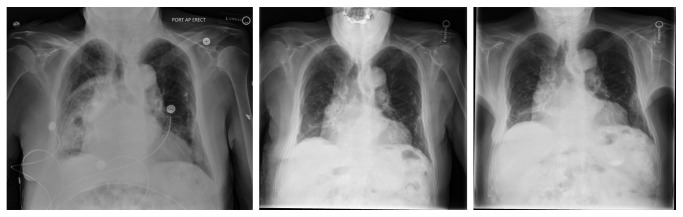
Chest X-rays from admission and at 1-month and 3-month follow-ups.

**Figure 2:**
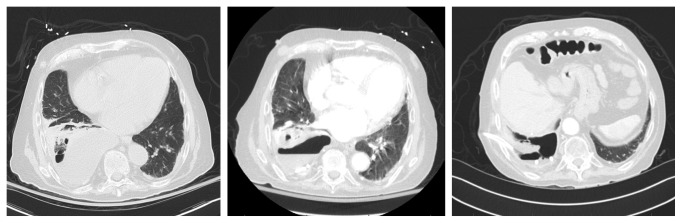
CT chest from admission, after chest tube placement, and at 1-month follow-up.

On arrival, the patient's WBC count was 12,500/*µ*l with 85.9% neutrophils. ABG showed pH of 7.496 with pCO_2_ 33.9 mmHg, pO_2_ 80.2 mmHg, and bicarbonate 26.4 meq/l. Chest X-ray revealed new, moderately extensive, multifocal right lung pneumonia with small associated parapneumonic effusion (Figure 1). CT chest further detailed a right basilar opacification with right pleural effusion and small amount of pleural gas (Figure 2). The patient was started on IV vancomycin, piperacillin-tazobactam, and oral azithromycin in the ED. Azithromycin was discontinued a day later after recommendations from Infectious Disease. Blood, sputum, and pleural fluid studies were ordered. Thoracocentesis showed pH 6.9, protein 4.6 g/dl (serum protein 5.7 g/dl; reference range: 6.4–8.9 g/dl), albumin 2.1 g/dl (serum albumin 2.2 g/dl), and lactate dehydrogenase (LDH) 4844 IU/L (serum LDH: 273 IU/L), suggestive of empyema. A 12 Fr chest tube was placed under CT guidance by interventional radiology which yielded foul-smelling, dark burgundy fluid. Pleural fluid culture was positive for pansensitive *C. perfringens*. Surprisingly, sputum culture grew *Pseudomonas aeruginosa* and *Citrobacter koseri*, both of which were sensitive to piperacillin-tazobactam. We did not order a quantitative culture to differentiate between pulmonary colonization and infection. We also did not perform bronchoscopy to rule out any mass or foreign body associated with the empyema. IV vancomycin was discontinued. IV clindamycin was added but was later discontinued after stool was positive for *Clostridium difficile*. The patient was planned for a 4-week course of IV piperacillin-tazobactam. On day 8, the patient was noted to have right lower extremity swelling which led to the discovery of extensive DVT and submassive PE, which was treated with Eliquis. Due to bleeding risk, decortication was deferred and the patient was instead treated with intrapleural tPA and dornase alpha.

With further questioning during hospital stay, the patient mentioned that he had difficulty swallowing food due to the sensation that something was stuck in his throat. He had resting tremor of the left hand and subtle cogwheeling of the right upper extremity. The swallowing function study was positive for aspiration. MRI brain to rule out any intracranial pathology was done which showed focal acute/subacute infarction. Echocardiogram with double bubble study revealed a patent foramen ovale. For this, cardiology advised anticoagulation with consideration for closure in the event of a stroke while on Eliquis. Neurology suspected that dysphagia was due to Parkinson's disease and recommended starting Sinemet in the near future. The patient was discharged to acute rehab with a chest tube to water seal. Chest CT done a month later showed improvement of the effusion with minimal drainage from the tube. The chest tube was removed. Repeat pleural fluid cultures were negative for infection. The patient did not show any fluid reaccumulation on subsequent imaging studies (Figures 1 and 2).

## 3. Discussion

Detection of pleuropulmonary infections with *C. perfringens* has improved with advances in sampling and culture methods [5]. Despite that, *C. perfringens* is rarely the cause of empyema [3]. Jackson et al. conducted a study for surveillance of invasive *C. perfringens* in 1 million residents in Alberta, Canada, but did not detect any case of *C. perfringens*-related empyema [6].

Clostridia are commonly found as commensals in the intestines and in soil [3, 7]. They can also colonize the skin especially with hospitalization [5]. Infection into the pleural space has been explained by various mechanisms. Entry of the infection can occur through open wounds or during invasive procedures like thoracocentesis, chest tube drainage, or surgery [3, 5, 7–9]. Underlying lung pathology like tuberculosis and pleural effusions and chronic diseases such as cirrhosis, diabetes, and malignancy increase predisposition [1, 5]. Aspiration has been described as a possible source of infection, as clostridia have been isolated in the oral flora of hospitalized patients [5, 8, 9]. Mixed organisms are usually seen in these patients [5]. Hematogeneous seeding of *C. perfringens* has been reported after sigmoid biopsy and esophageal rupture [3, 5]. Bashir et al. reported cases of necrotizing pneumonia complicating pulmonary embolus [4].

Our patient may have acquired *C. perfringens* during his recent hospitalization. With his swallowing dysfunction, he may have aspirated the organism. Surprisingly, anaerobic sputum cultures failed to detect *C. perfringens*. Hematogeneous seeding is a possibility in our case, but blood cultures were negative. Bashir et al. reported *C. perfringens* complicating pulmonary emboli, but they thought that these two entities were unrelated [4]. Although our patient had pulmonary embolus, it was absent when infection was first detected. Kwan et al. reported *C. perfringens* empyema without pneumonia [3]. Our patient had pneumonia with parapneumonic effusion, but the organisms cultured were different. The pleural fluid is classically malodorous, dark red to brown as in our patient [2]. Imaging shows air-fluid levels with gas production which can be confused with herniation of the intestine into the thoracic cavity [2, 5]. The mainstay of management is surgical drainage and antibiotics [2, 3, 8, 9]. Penicillins are the first-line choice [2]. Use of another antibiotic like clindamycin, metronidazole, or chloramphenicol is indicated only in cases of penicillin allergy [2, 5]. Roberts et al. reported 100% susceptibility to penicillins (amoxicillin/clavulanic acid and piperacillin-tazobactam), cephalosporins (cefoxitin, cefotetan, and ceftriaxone), clindamycin, carbapenems (imipenem and meropenem), and metronidazole [10]. The role of antitoxins and hyperbaric oxygen therapy has not yet been established [2, 3].

## 4. Conclusion

Prognosis for *C. perfringens* is good with appropriate treatment including pleural drainage and appropriate antianaerobic antibiotic therapy [9]. Clinicians must be aware of this etiology for pleural infections for prompt treatment.
